# Retained fetal bone post‐abortion causing infertility

**DOI:** 10.1002/ccr3.5966

**Published:** 2022-06-21

**Authors:** Jiexin Cao, Carla‐Marie Grubb, Mian Khurshid, Aparna Gumma

**Affiliations:** ^1^ Obstetrics and Gynaecology Department Ysbyty Gwynedd Bangor UK

**Keywords:** abortion, infertility, retained fetal bone

## Abstract

Fetal bone retention is a rare but under‐diagnosed complication after abortion. If left untreated, it can cause menstrual dysfunction and secondary infertility. We present a case of a 39‐year‐old woman who undergone abortion 20 years ago but suffered with secondary infertility due to retained fetal bone.

## Abstract 2

Retained fetal bone is a rare complication for abortion and can cause secondary infertility. There is currently no comprehensive guidance in abortion care in the UK regarding this complication, hence we call for awareness and development of guideline.

## 1 INTRODUCTION

Retained fetal bone fragments are a rare complication of abortion and can be a cause of secondary infertility.^1^ No evidence for clinical signs of retained fetal bone fragments is widely available, but they are thought to include dysmenorrhea and other menstrual irregularities, chronic pelvic pain, and secondary infertility.^2^ No agreed‐upon protocol or guidelines regarding the best management are currently available, but most case reports and case series discuss patients who have been managed by hysteroscopic retrieval of the fetal bone fragments. We present a case of secondary infertility likely caused by retained fetal bone fragments and its diagnostic challenges. We also discuss current guidelines in the diagnosis and management of such scenarios.

## 2 CASE HISTORY

**FIGURE 1:**
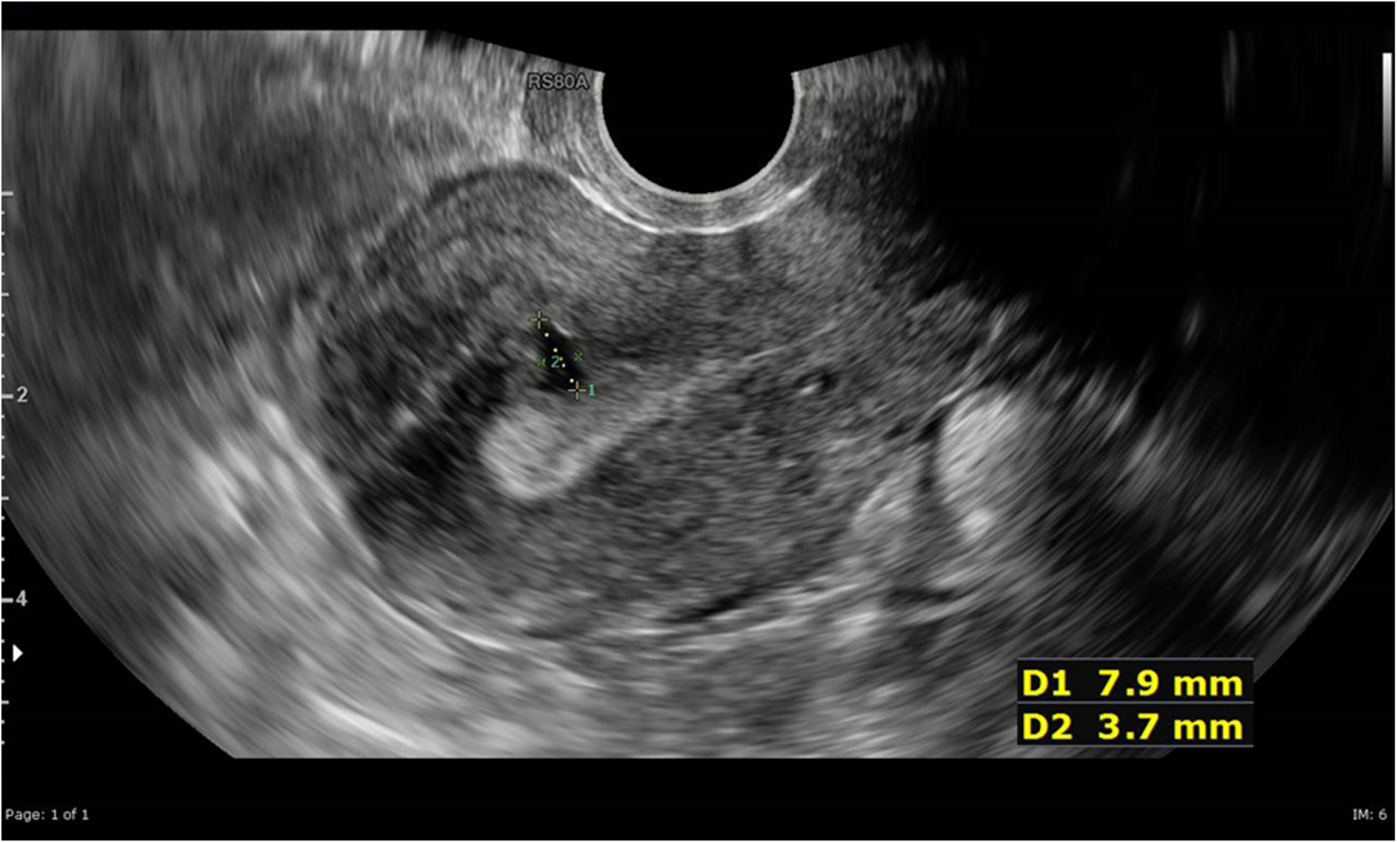
Transvaginal ultrasound showing echotexutre at the fundus

A 39‐year‐old female patient was initially seen in 2018 in a general gynecology clinic with complaints of chronic pelvic pain and 2 years of infertility. Her pain was non‐cyclical, spasmodic, and often had a sudden onset. She had recurrent herpes infections for which she took acyclovir when needed, but she was otherwise fit and well. She had no history of drug allergy. She was an active smoker, smoking 1–2 cigarettes/day, and she drank 20 units of alcohol per week. Her body mass index was 31.8. A previous obstetric history revealed a mid‐trimester abortion 20 years ago because she was not mentally and physically ready to carry on with the pregnancy. The abortion was performed with surgical dilatation and curettage at 20 weeks. Transvaginal ultrasound investigation showed heterogeneous echotexture in the anterior fundal wall containing a cystic area 8 mm in size (Figure 1). The initial diagnosis from the clinic was irritable bowel syndrome; however, adenomyosis needed to be ruled out. Therefore, magnetic resonance imaging (MRI) scans were booked to further investigate, and mebeverine was prescribed.

**FIGURE 2:**
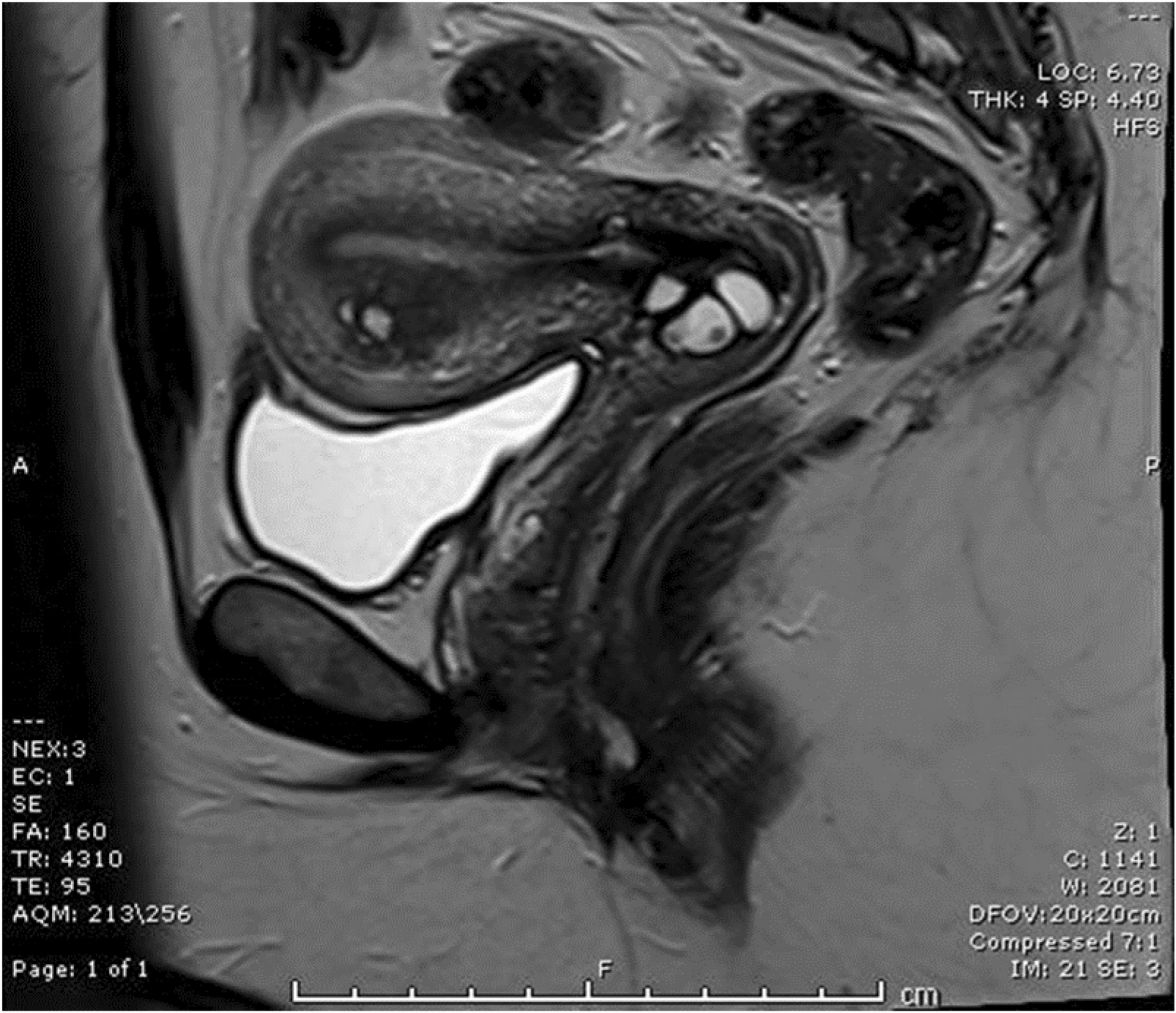
MRI scan showing fundal cystic structure

The patient was seen again in the gynecology outpatient clinic in 2019. The MRI showed a small fundal cystic structure (Figure 2). A second ultrasound showed ovulation. She was then referred to the infertility clinic. The semen analysis was normal, and the chlamydia and smear tests were negative. Hysterosalpingography was organized but unable to cannulate the cervix. The patient was therefore referred to the tertiary fertility center.

Unfortunately, the tertiary referral was declined because both she and her partner were active smokers. Smoking cessation advice was given.

**FIGURE 3:**
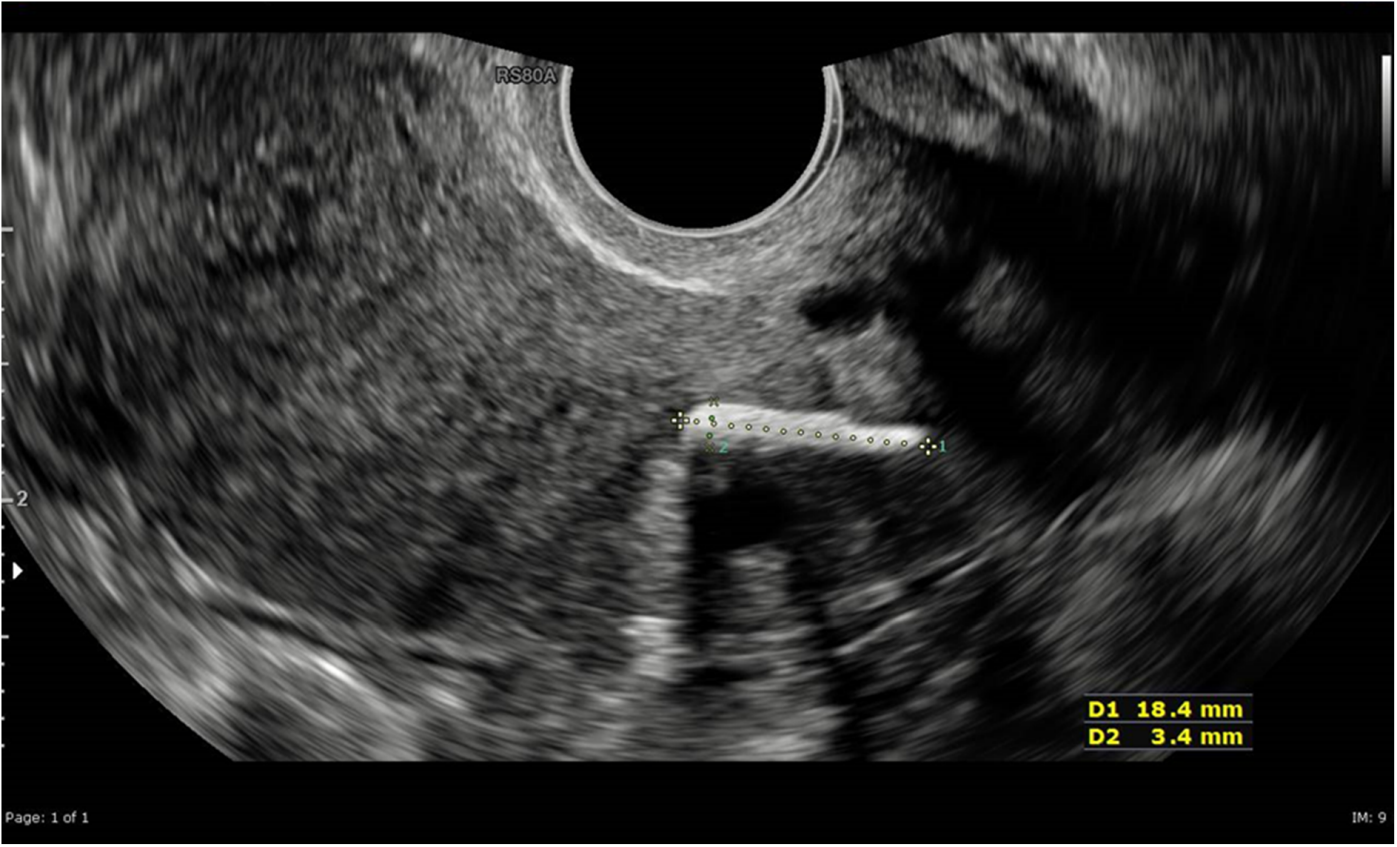
Transvaginal ultrasound showing possible foreign body

In 2020, the patient once again presented to the general gynecology clinic with chronic pelvic pain on the left side, dysmenorrhea, and menorrhagia. She was booked for diagnostic laparoscopy and dye tests. While waiting for the operation, the patient underwent repeat pelvic ultrasound, which showed a linear, highly reflective foreign body of 18 × 3 mm within the endometrial cavity and adjacent to the cervix (Figure 3). Evidence of adenomyosis was noted, and the endometrium appeared normal.

Hysteroscopy and diagnostic laparoscopy with a dye test were performed in 2021. The cervix was noted to be pin‐hole sized and very difficult to dilate. A foreign body was observed and removed from the endometrial cavity; the endometrial cavity otherwise looked normal. Laparoscopy revealed left‐sided physiological adhesion of the bowel to the pelvic side wall. The left tube looked inflamed, but the dye test was positive for both tubes. Both ovaries appeared normal. Histology of the foreign body showed fragments of dead bone.

## 3 DISCUSSION

This case highlights the difficulty in diagnosing the cause of secondary infertility and abdominal discomfort by noting that the first ultrasound scan as well as the MRI scan did not show any foreign bodies inside the endometrium. Potential differential diagnoses may include osseous metaplasia driven by chronic inflammation causing dystrophic calcification,^3^ endometrial ossification driven by chronic inflammation, and tissue destruction in the mature endometrial stroma.^4^ These two differential diagnoses can be ruled out because the segment retrieved by hysteroscopy looked like a fetal long bone, and the histology showed dead bone fragments but no active tissue.

Fetal bone fragments residing in the endometrium can act as foreign bodies, causing chronic inflammation in the endometrial environment, inhibiting implantation of the embryos, and causing secondary infertility. This effect is similar to that of an Intrauterine Contraceptive Device (IUCD) in situ. Some research has suggested that the fetal bone may have a direct toxic effect on the developing embryo, thereby causing infertility.^4^

Currently, hysteroscopy is used in most cases as the treatment of choice to retrieve a foreign body to treat infertility^1^, ^2^, ^3^, ^4^ or menstrual complaints, including chronic pelvic pain.^5^ However, there is a lack of national guidance regarding gold standard investigations or treatments. In fact, there is a lack of evidence surrounding the follow‐up of abortion care in general. The National Institute for Health and Care Excellence (NICE) guidelines,^6^ as well as the best practices in abortion care provided by the college,^7^ do not provide specific guidance regarding symptoms and signs of incomplete abortion, especially post‐mid‐trimester abortion. Guidance on how to investigate suspected incomplete abortions is also lacking. Because more than 70% of abortions are performed in the private sector,^6^ it is understandably difficult to follow‐up with patients and to communicate with the primary and secondary care providers regarding their treatments. However, as retained fetal tissue and bones can cause a number of menstrual and fertility problems in women, it is important to obtain a detailed obstetric history, including any abortions, and bear in mind such a differential diagnosis. Often, a hidden history of mid‐trimester abortion can guide the interpretation of vague clinical symptoms and uncertain echogenicities in the endometrium seen on ultrasound scans. The current guidelines should also be reviewed for guidance in terms of early recognition, investigation, and appropriate treatment and follow‐up for these women, especially when the abortion rate is on the rise with the current pandemic.^8^

## AUTHOR CONTRIBUTIONS

Dr J Cao has participated in the care of the patient, researched and written the case report. Dr A Gumma has participated in the care of the patient, initiated and reviewed the case report. Dr C Grub has participated in the care of the patient and reviewed the case report. Mr M Khurshid has participated in the care of the patient.

## CONFLICT OF INTEREST

None.

## CONSENT

Written informed consent was obtained from the patient to publish this report in accordance with the journal's patient consent policy.

## Data Availability

Data sharing is not applicable to this article as no new data were created or analyzed in this study.
